# Population Genetic Structure and Chemotype Diversity of *Fusarium graminearum* Populations from Wheat in Canada and North Eastern United States

**DOI:** 10.3390/toxins13030180

**Published:** 2021-03-01

**Authors:** Abbot O. Oghenekaro, Maria A. Oviedo-Ludena, Mitra Serajazari, Xiben Wang, Maria A. Henriquez, Nancy G. Wenner, Gretchen A. Kuldau, Alireza Navabi, Hadley R. Kutcher, W. G. Dilantha Fernando

**Affiliations:** 1 Department of Plant Science, University of Manitoba, 66 Dafoe Road, Winnipeg, MB R3T 2N2, Canada; abbot.oghenekaro@umanitoba.ca; 2 Department of Plant Sciences, University of Saskatchewan, 51 Campus Drive, Saskatoon, SK S7N 5A8, Canada; mao722@mail.usask.ca (M.A.O.-L.); randy.kutcher@usask.ca (H.R.K.); 3 Department of Plant Agriculture, University of Guelph, 50 Stone Road E Guelph, Guelph, ON N1G 2W1, Canada; mserajaz@uoguelph.ca; 4 Agriculture and Agri-Food Canada (AAFC), Morden Research and Development Centre, 101 Route 100, Morden, MB R6M 1Y5, Canada; xiben.wang@canada.ca (X.W.); mariaantonia.henriquez@canada.ca (M.A.H.); 5 Department of Plant Pathology and Environmental Microbiology, Pennsylvania State University, 211 Buckhout Lab, University Park, PA 16802, USA; ngw1@psu.edu (N.G.W.); kuldau@psu.edu (G.A.K.)

**Keywords:** *Fusarium graminearum*, trichothecene, population genetics, 3ADON, 15ADON

## Abstract

Fusarium head blight (FHB) is a major disease in wheat causing severe economic losses globally by reducing yield and contaminating grain with mycotoxins. In Canada, *Fusarium graminearum* is the principal etiological agent of FHB in wheat, producing mainly the trichothecene mycotoxin, deoxynivalenol (DON) and its acetyl derivatives (15-acetyl deoxynivalenol (15ADON) and 3-acetyl deoxynivalenol (3ADON)). Understanding the population biology of *F. graminearum* such as the genetic variability, as well as mycotoxin chemotype diversity among isolates is important in developing sustainable disease management tools. In this study, 570 *F. graminearum* isolates collected from commercial wheat crops in five geographic regions in three provinces in Canada in 2018 and 2019 were analyzed for population diversity and structure using 10 variable number of tandem repeats (VNTR) markers. A subset of isolates collected from the north-eastern United States was also included for comparative analysis. About 75% of the isolates collected in the Canadian provinces of Saskatchewan and Manitoba were 3ADON indicating a 6-fold increase in Saskatchewan and a 2.5-fold increase in Manitoba within the past 15 years. All isolates from Ontario and those collected from the United States were 15ADON and isolates had a similar population structure. There was high gene diversity (*H* = 0.803–0.893) in the *F. graminearum* populations in all regions. Gene flow was high between Saskatchewan and Manitoba (*Nm* = 4.971–21.750), indicating no genetic differentiation between these regions. In contrast, less gene flow was observed among the western provinces and Ontario (*Nm* = 3.829–9.756) and USA isolates ((*Nm* = 2.803–6.150). However, Bayesian clustering model analyses of trichothecene chemotype subpopulations divided the populations into two clusters, which was correlated with trichothecene types. Additionally, population cluster analysis revealed there was more admixture of isolates among isolates of the 3ADON chemotypes than among the 15ADON chemotype, an observation that could play a role in the increased virulence of *F. graminearum*. Understanding the population genetic structure and mycotoxin chemotype variations of the pathogen will assist in developing FHB resistant wheat cultivars and in mycotoxin risk assessment in Canada.

## 1. Introduction

Fusarium head blight (FHB) is a global disease affecting small grain cereals including wheat, barley and oat [1,2,3,4] The disease is caused by several *Fusarium* species of which *Fusarium graminearum* Schwabe is the primary etiological agent in wheat crops in Canada based on the frequency of isolation and impact on grain yield and quality [5,6,7,8]. In addition to a reduction in yield, *F. graminearum* produces the type B trichothecene mycotoxins, deoxynivalenol (DON) and its acetylated derivatives (15-acetyl-DON (15ADON) and 3-acetyl-DON (3ADON)) in Canada [7,9]. The contamination of wheat by mycotoxins is a serious health concern for both humans and animals. This has led to strict DON tolerance limits in Canada that are established at 2 mg kg^−1^ body weight for unprocessed grain and 0.2 mg kg^−1^ for baby food [10].

Epidemics of FHB in western Canada started in the early 1980s when grain samples of wheat from Manitoba showed symptoms of bleached kernels and detectable levels of DON mycotoxin [11,12]. Ever since, increasingly frequent FHB epidemic years occurred in Canadian prairies with the worst outbreak in 2016 in Saskatchewan (SK) and Manitoba (MB) [13]. More recently, outbreaks have extended to the Province of Alberta. Comprehensive surveys of MB and SK wheat fields in the last three years have shown an FHB prevalence (proportion of fields affected) ranging from mild to very serious. One of the most recent epidemics occurred in 2016 with 87% FHB prevalence among wheat fields in Manitoba [8,14,15]. High precipitation, relative humidity and warm temperatures during and after anthesis favors FHB development in wheat [16]. Epidemics of FHB is also enhanced by rapid evolution of *Fusarium* pathogens and the ability of the disease to spread through airborne ascospores over large areas [17]. These important factors increase the risk of the development of FHB epidemics in the future.

In Canada, FHB outbreaks lead to serious losses in terms of yield coupled with grain contaminated by mycotoxins. Moderately resistant FHB cultivars have been developed through breeding efforts and are used extensively in western Canada; these include AAC Brandon, Cardale and Carberry [18] with AAC Brandon being the predominant cultivar with 66.2% of the acreage in Manitoba in 2019 [19]. Despite the moderate resistance achieved in many cultivars, the percentage of diseased spikes can increase dramatically in severe epidemics. Several surveys and molecular mycotoxin profiling over the last 20 years have shown a temporal and spatial increase in the 3ADON isolates compared to the 15ADON isolates, with a 3ADON vs 15ADON frequency of 10.7% and 31% 3ADON in Saskatchewan and Manitoba, respectively, between 1984 and 2004 [7,9,20]. However, these studies did not consider the mycotoxin sub-structuring profile within the geographic locations in the provinces in Canada.

Population biology studies are critical in providing information that can be utilized to predict disease epidemics and also assist in setting up trials in developing FHB resistant cultivars. Genetic variation in a population is a strong indicator of frequent sexual reproduction and the rapid evolution of a pathogen. Such populations with high genetic variation can quickly adapt to resistant cultivars and fungicide modes of action [21]. The ability of genes or genotypes to migrate to new geographic areas can be predicted by the amount of gene flow between populations [22]. Surveys of *Fusarium* species associated with FHB in wheat across Canada have shown *F. graminearum* to be predominant at up to 90% of the *Fusarium* spp. [7,23,24]. Population genetic studies of *F. graminearum* collected from wheat in Canada are few and either limited to a single province or performed with isolates collected about 15 years ago. A large-scale population structure study of Canadian isolates collected in 2005–2007 was investigated by [7] revealing a one-fold increase in the 3ADON populations in 2005–2007. Population genetic studies in Manitoba was carried out on isolates collected in 2004–2005 [23,25]. A more recent study in the Province of Ontario utilized isolates collected in 2010–2012 [24]. Pathogen populations can be dynamic in terms of evolutionary rates over space and time, and coupled with changing agronomic practices, can have a huge influence on pathogen population dynamics in a particular geographic location. It is therefore necessary to monitor *F. graminearum* populations in wheat to detect changes in population structure and understand how these changes can influence the proliferation and development of FHB.

Molecular markers that are unambiguous and polymorphic are important tools in the study of the population biology of plant pathogens [21]. Several genetic markers have been utilized to study genetic diversity in *F. graminearum* populations. These include random amplified polymorphic DNA, restriction fragment length polymorphism, amplified fragment length polymorphism, microsatellite or simple-sequence repeats and sequence-related amplified polymorphisms [23,26,27,28,29]. For this study, we used the variable number of tandem repeats (VNTR) developed by [30] from the genome of *F. graminearum* PH−1 (NRRL31084). These markers are more suited to genetic diversity studies than other markers because of the simplicity of polymorphic data collection [30].

We hypothesize that the mycotoxin chemotype pattern of *F. graminearum* in wheat is reflected in the population structure within and between provinces in western Canada. The specific objectives were to: (i) investigate the genetic variability and population structure of *F. graminearum*, and the patterns of relatedness of the populations in Manitoba and Saskatchewan, (ii) investigate the level of gene flow and to determine the correlation of genetic distance with genetic identity, and (iii) determine the DON chemotype distribution within and between the provinces and their relationship with population genetic structure.

## 2. Results

### 2.1. Sample Collections

**Figure 1:**
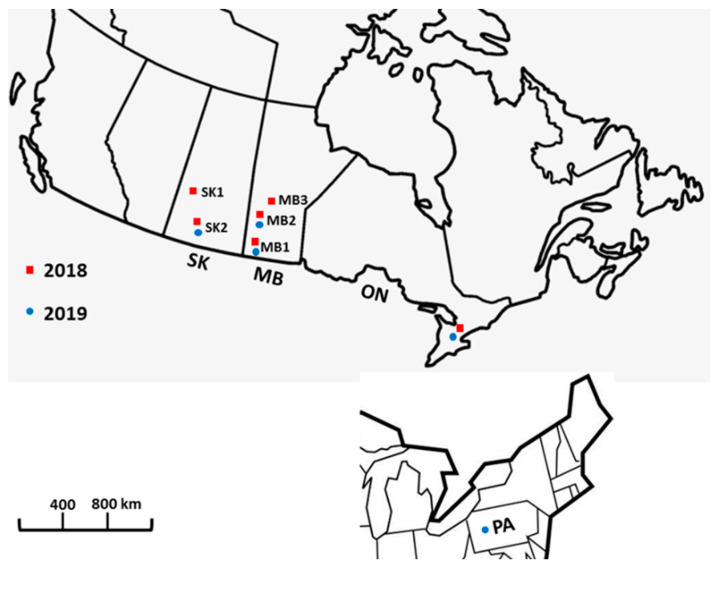
Map of Canada and the State of Pennsylvania, USA showing the geographic regions sampled in 2018 and 2019. SK—Saskatchewan (SK1—Parkland region, Saskatchewan, SK2—southern Saskatchewan) MB—Manitoba (MB1—southwest Manitoba, MB2—central Manitoba, MB3—Interlake region, Manitoba) ON—Ontario, PA—Pennsylvania, USA.

Three provinces in Canada (Saskatchewan, Manitoba and Ontario) were sampled in 2018 and 2019. These includes two geographic regions in Saskatchewan and three in Manitoba. A subset of isolates from The State of Pennsylvania in northeastern USA was also added to the analysis. In total, 570 isolates of *F. graminearum* were recovered and used for genetic structure analysis and chemotype diversity (Figure 1).

### 2.2. Trichothecene Chemotype Profiling of F. graminearum

**Figure 2:**
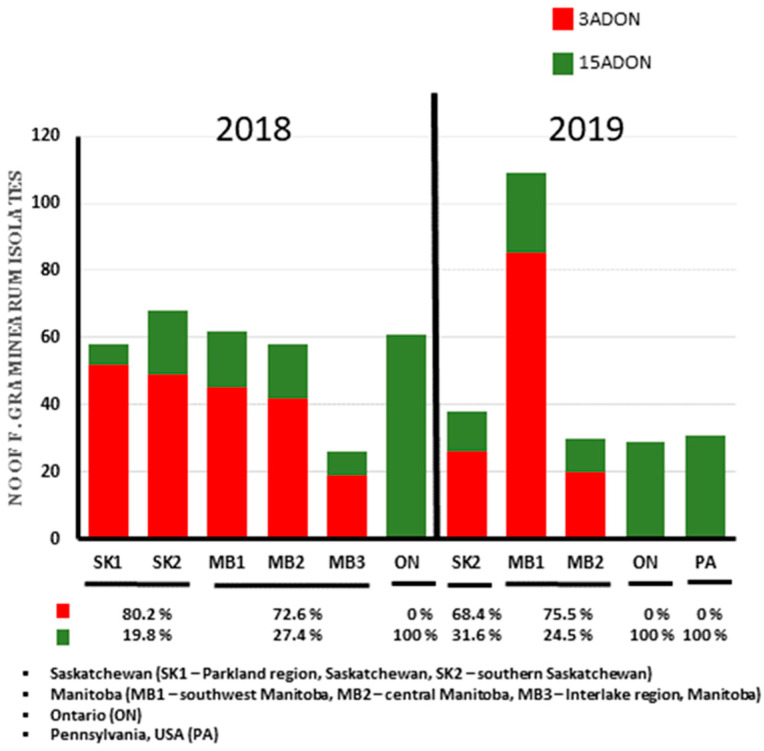
Distribution of 3-acetyl-deoxynivalenol (3ADON) and 15-acetyl-deoxynivalenol (15ADON) chemotypes among 570 *Fusarium graminearum* isolates from wheat.

Trichothecene chemotype prediction using multiplex PCR primers (3CON, 3NA, 3D3A and 3D15A) identified the 570 *F. graminearum* isolates collected in this study as DON chemotypes; either the 3ADON or 15ADON genotype. The 3ADON chemotypes predominated in Saskatchewan and Manitoba in both 2018 and 2019 (Figure 2, Table S1). The frequency of 3ADON chemotypes was >70% in all five geographic regions in western Canada (two in SK and three in MB) except in southern SK in 2019 where it was 68.4%. The frequency of 3ADON chemotypes at the provincial level was 80.2% and 68.4% in 2018 and 2019 for SK and 72.6% and 75.5% for MB (Figure 2, Table S1). The 91 isolates from Ontario (61 in 2018 and 30 in 2019) and 31 isolates from Pennsylvania, USA had 100% 15ADON composition (Figure 2, Table S1). In 2018, 40 out of 41 isolates from Melfort (SK1) and 35 out of 36 isolates from Indian Head (SK2), were of the 3ADON chemotype (Table S1). In some locations in MB, all isolates were 3ADON: 14 collected from Elton and North Cypress in Crop District 2 and 10 isolates from McDonald in Crop District 7 (Table S1).

### 2.3. Population Genetic Structure Analyses of Geographic Regions

The 10 VNTR markers utilized for genotyping the *F. graminearum* isolates were highly polymorphic in all geographic regions. The amplicon size ranged from 120–366 bp, while the allelic frequencies ranged from 0.001–0.302 (Table 1). The number of amplified alleles per locus ranged from 14 (HK913, HK917, HK967) to 31 (HK957) making the VNTR locus, HK957 located on chromosome 1, the most polymorphic marker (Table 1). 

The genetic diversity statistics of the seven geographic regions were analyzed with GenAlEx. There were 562 unique multilocus haplotypes (98.6% haplotype diversity) in all samples (Table 2). Shannon’s diversity indices (*I*) were high for all geographic regions (Table 2), ranging from 1.834 (Parkland region of SK) to 2.390 (central MB). High genetic diversity was found in all geographic regions studied. The lowest and highest unbiased gene diversities (*uH*) were also found in the Parkland region of SK, (0.803) and central MB (0.893), respectively. The number of effective alleles (*N_e_*) within a population ranged from 5.692 in PA to 9.679 in central MB (Table 2).

Pairwise comparisons of genetic identity across the seven geographic regions ranged from 0.416 (MB3 and PA) to 0.889 (SK2 and MB1). Genetic distance pairwise comparisons ranged from 0.118 (SK2 and MB1) to 0.877 (MB2 and PA) (Table 3).

The estimated pairwise *F_ST_* values between the geographic regions were generally very low between the regions in western Canada, but statistically high when compared to the ON and PA isolates. It ranged from 0.011 (SK2 and MB1/MB1 and MB2) to 0.082 (MB3 and PA). The southwest MB and central MB population pair showed the highest gene flow (*Nm* = 21.750), while the lowest gene flow (*Nm* = 4.971) was recorded between the interlake region of MB and PA (Table 4).

The analysis of molecular variance using GenAlEx showed that a large proportion (96%) of the observed genetic variance was explained by allelic variation within the geographic regions and 4% between geographic regions (Table 5).

**Figure 3:**
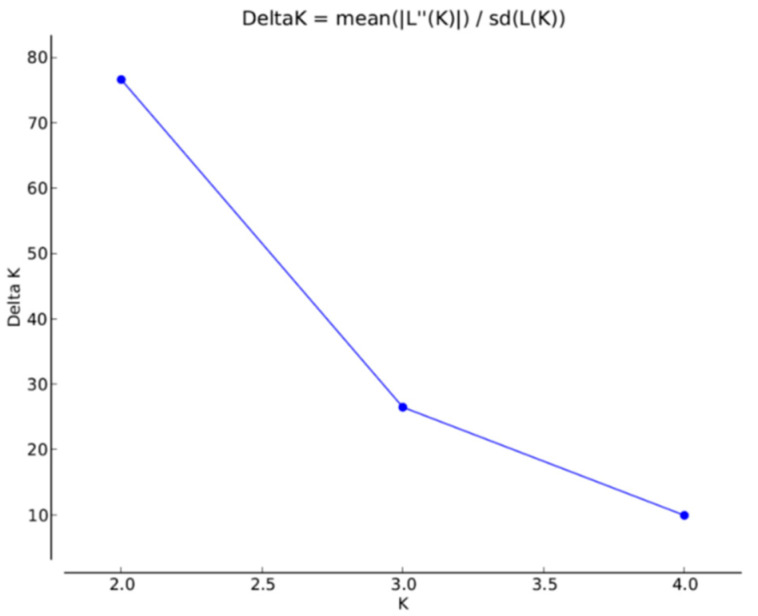
Δ*K* calculated by the Evanno method [33] showing the *K* value that had the highest rate of change in log likelihood, here *K* = 2. The test was performed with VNTR data of all 562 clone corrected *Fusarium graminearum* isolates with the number of simulated populations (*K*) ranging from 1 to 5, with 10 replications for each *K* value.

**Figure 4:**
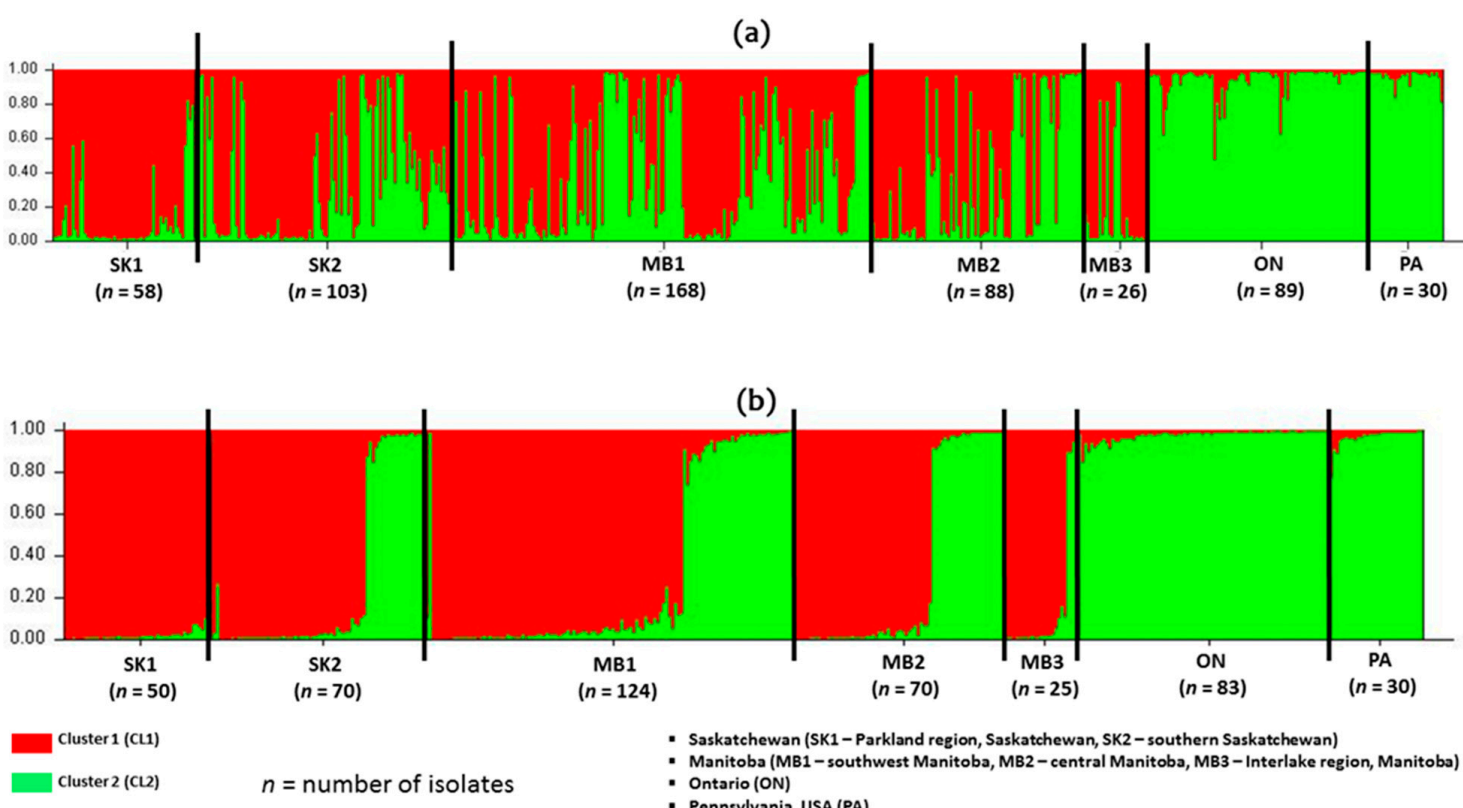
Population structure and admixture estimates of 562 clone corrected *Fusarium graminearum* isolates based on geographic locations (**a**) All 562 isolates (**b**) Isolates with membership proportion greater than 0.8 for both CL1 and CL2. Vertical bars represent individual isolates colored to represent the estimated proportion of K membership of each isolate, which ranged from 0 to 1. Each bar represents one isolate.

The STRUCTURE program was utilized to analyze and identify possible structuring of subpopulations as geographic regions among the *F. graminearum* isolates. The highest rate of change in log-likelihood values was obtained at *K* = 2, Δ*K* (>75). Values of Δ*K* for all other *K* tested was <30 (Figure 3). Thus, dividing the isolates into two genetic clusters captured the major genetic structure in the data (Figure 4). Assignment of the 562 clone-corrected *F. graminearum* isolates into the two clusters, designated as CL1 and CL2 based on the Bayesian model-based structuring implemented in the program STRUCTURE is shown in Table 6. The proportion of membership (*q*), of each cluster, ranging from 0 to 1, for each isolate in the seven populations was determined and the isolates were assigned to the cluster with the highest proportion. The number of isolates assigned to the two clusters was 309 (55%) for CL1 and 253 (45%) for CL2 (Table 7). The clusters, CL1 (Red) and CL2 (green) contained isolates from all geographic regions except Ontario and Pennsylvania, which had quite all isolates from CL2. A higher percentage of isolates from the five western Canadian regions (SK1, SK2, MB1, MB2 and MB3) belong to the CL1 cluster, which indicated no genetic structuring among these regions (Table 6). In contrast and interestingly, all isolates from Ontario, eastern Canada and PA eastern USA belong to the CL2 cluster indicating no genetic structuring between these two regions (Table 6). The cluster grouping indicated that populations in western Canada are different from those in eastern Canada and the USA. Population structure and admixture estimates of all the isolates for each cluster are shown in Figure 4a indicating no genetic structure between Saskatchewan and Manitoba isolates, but a clear genetic structure if compared to the Ontario and Pennsylvania populations. Further proof of this structuring was observed if the analysis is done with only isolates with membership proportion greater than 0.8 for both CL1 and CL2 (Figure 4b).

### 2.4. Population Genetic Structure Analyses of Chemotype Populations 

**Figure 5:**
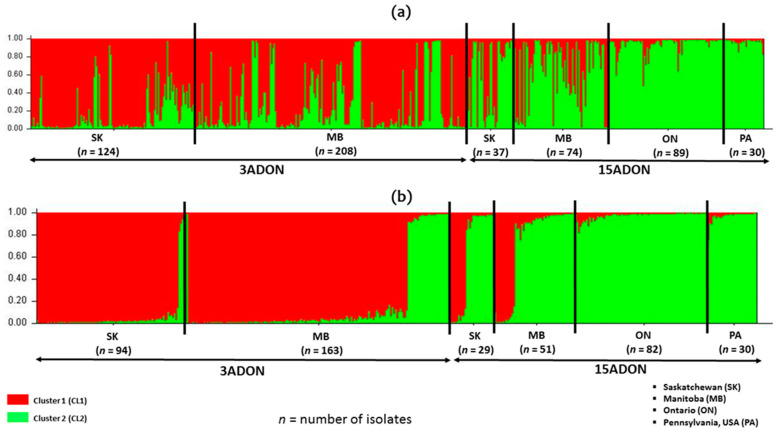
Population structure and admixture estimates of 562 clone corrected *Fusarium graminearum* isolates based on chemotypes; 3ADON and 15ADON (**a**) All 562 isolates (**b**) Isolates with membership proportion greater than 0.8 for both CL1 and CL2. Vertical bars represent individual isolates colored to represent the estimated proportion of K membership of each isolate, which ranged from 0 to 1. Each bar represents one isolate.

The chemotypes were also assigned to the clusters generated from STRUCTURE analysis. Both clusters (CL1 and CL2) had high Shannon Information index (*I*) and gene diversity (*H*) of 2.221 and 2.356 and 0.859 and 0.880, respectively (Table 7). Cluster 1 (CL1) had a higher proportion of the 3ADON chemotype (77.4%), while cluster 2 had a higher proportion of the 15ADON (63.2%). Population structure and admixture estimates of the isolates were grouped according to chemotypes and analyzed with STRUCTURE (Figure 5). Interestingly, the 3ADON and 15ADON isolates from Saskatchewan, Manitoba, Ontario and Pennsylvania showed a genetic structuring which grouped according to chemotype regardless of geographic origin, indicating a strong correlation to trichothecene type (Figure 5a). Interestingly, a higher number of admixture isolates (isolates with close to equal coefficient of membership of CL1 and CL2) was observed among the 3ADON isolates compared to the 15ADON (Figure 5a). Further proof of this chemotype structuring was detected when the analysis was done on isolates with membership proportion greater than 0.8 for both CL1 and CL2 (Figure 5b).

## 3. Discussion

*Fusarium graminearum* is an economically important plant pathogenic fungus because it is the primary causal agent of FHB in small grain cereals. In Canada, it causes substantial damage to wheat. The optimum conditions for the proliferation of FHB are warm temperatures and high (>90%) relative humidity during flowering [34]. This is a strong indication that the pathogen will continue to be a subject of interest in the future due to the impact of climate change and global warming [35]. In the present study, we used VNTR markers to analyze and characterize the population genetic diversity and structure of *F. graminearum* isolates collected in three provinces in Canada and in the northeastern USA.

The *F. graminearum* VNTR markers have been widely used in *F. graminearum* population genetic analysis [7,9,24,36,37,38,39,40,41,42,43]. In the current study, a high level of polymorphism was detected as indicated by the relatively high Shannon Index values across the geographic regions. This was also identified in the ten VNTR markers used in this study, which proved that it is an appropriate type of marker system for population genetic studies of *F. graminearum*. The high polymorphism and unique haplotypes (98.6%) identified showed the high genetic diversity and very limited clonal populations of *F. graminearum* in wheat in Canada.

The present work is the most recent wide-scale study of *F. graminearum* population structure in Canada. Previous population genetic analysis of *F. graminearum* in Canada has been done on isolates collected in 2001–2008 [7,9,23]. Our study indicates very high gene flow among isolates from different geographic regions in the two neighboring western provinces: Saskatchewan and Manitoba. The high gene flow points to subpopulations within the two provinces that are part of a larger population that allows for frequent random mating. High gene flow between the eastern Canadian province of Ontario and PA in northeast USA also indicates they probably belong to one population. However, some level of genetic structure was observed for the two trichothecene chemotypes (3ADON and 15ADON) irrespective of geographical locations. This is in agreement with previous results of the clustering of 3ADON and 15ADON chemotypes across Canada [7,9] and the USA [27].

Previous reports have shown that the majority of *F. graminearum* isolates in wheat in Canada exhibit two longitudinal clines explaining the distribution of the 3ADON and 15ADON chemotypes. The 15ADON chemotype is predominant in eastern Canada (Ontario and Quebec) where the ratio of 15ADON to 3ADON remained relatively constant over the last 15–20 years [7,24]. In contrast, the 3ADON chemotype is predominant in the western provinces of Canada (Alberta, Saskatchewan and Manitoba) and there has been a temporal and spatial increase in 3ADON isolates over the last 20 years [7,9]. Results from this study support the temporal increase in 3ADON chemotypes in western Canada as over 70% of the isolates were of the 3ADON genotypes. Ref. [9] reported a 10.7% and 31% 3ADON chemotype percentage in 2004 for Saskatchewan and Manitoba. Breakdown of the results of this study show that the 3ADON chemotype increased in Saskatchewan more than 6-fold between 2004 (10.7%) and 2019 (68.4%) and increase in Manitoba 3ADON more than 2.5-fold between 2004 (31%) and 2019 (75.5%). Ref. [23] reported 33.7% 3ADON isolates from 291 isolates of *F. graminearum*, while the 3ADON percentage in this study of 570 isolates was above ≥70% in both 2018 and 2019. In addition, two regions sampled by [23] from Manitoba (Towns of Killarney and Cartier), which had ≥72% 15ADON composition had a chemotype shift that is now ≥75% 3ADON as recorded in this study. The exact reason for this chemotype shift is a subject of debate but may be due to adaptation as suggested by [9], as the 3ADON isolates have been reported to be more aggressive than their 15ADON counterparts. The higher admixture level observed in the 3ADON isolates might play a role in its higher virulence compared to the 15 ADON isolates [39]. Fungal pathogens with high admixture can significantly influence and speed the evolution of virulence [44].

The chemotype composition of *F. graminearum* in western Canada was the opposite of that observed in eastern Canada. The 15ADON chemotype is predominant in eastern Canada and PA in the United States. Ref. [24] and [45], respectively, reported a 98% and 97% 15ADON composition of *F. graminearum* isolates collected from wheat in the Province of Ontario in 2008–2013. After extensive sampling and chemotyping in the mid-west United States, researchers have reported a high 15ADON composition [46,47,48]. Interestingly in this study, all isolates collected from wheat in Ontario and Pennsylvania (northeastern USA) were of the 15ADON chemotype. The high proportion of 3ADON isolates recorded in both Saskatchewan and Manitoba in this study emphasizes the need for regular temporal and spatial monitoring of trichothecene chemotypes across Canada. In addition, Bayesian model population structure analysis identified a different population structure that is associated with trichothecene chemotype differences. This study as well as previous studies on chemotype populations have demonstrated a significant temporal chemotype change in western Canada, which might be strongly linked to differences in local selective pressures among provinces in Canada. This further emphasizes the need for regional solutions to FHB management.

Spatial variability and relatedness of populations from the seven geographic regions were inferred from the genetic identity and genetic distance. Our population genetics analysis suggested that the five *F. graminearum* populations (SK1, SK2, MB1, MB2 and MB3) from western Canada were genetically similar. This is mainly supported by the high gene flow (*Nm*, 4.971–21.750) and low genetic differentiation (*F_ST_*, 0.011–0.048) among regions. Lower gene flow and relatively high genetic differentiation (*F_ST_*, 0.026–0.082) between the populations in the western provinces and that in Ontario and PA indicated a genetic structure between eastern and western regions. A similar correlation between genetic distance/identity and geographic location was reported for *F. graminearum* isolates collected from Alberta, Saskatchewan and Manitoba [49]. Ref. [23] also found high gene flow (*Nm* = 11.176) among 15 subpopulations of *F. graminearum* in Manitoba. Similarly, high gene flow (*Nm* = 19.483) was reported among *F. graminearum* populations collected in wheat fields in Alberta, Saskatchewan and Manitoba [49]. Similar high gene was also recorded between populations from central and southwestern Ontario [24]. The AMOVA results buttressed the points above, since very high genetic variation (96%) was found among individuals within the subpopulations compared with a very low genetic variation (4%) among the subpopulations. This suggests a greater probability of sexual reproduction among the *F. graminearum* populations in the regions studied. There was no significant difference in the population structure of the geographic regions studied, which agrees with previous studies of *F. graminearum* isolates collected across Manitoba using sequence-related amplified polymorphism [23]. Trade-in wheat seed between the two provinces is likely a major contributor to the high gene flow between isolates from the different geographic regions. Long-distance spore transfer as shown for *F. graminearum* [50] might also be a factor responsible for the high gene flow.

The 3ADON and 15ADON trichothecene chemotype populations from this study appear to be homogenous for chemotype and genetically similar with STRUCTURE Bayesian modelling analysis of cluster isolates. Homogeneity is more visible in the 15ADON chemotype isolates than the 3ADON chemotype isolates. Ref. [51] reported a similar homogenous grouping of 15ADON isolates from Canada, but the main reason for this homogeneity is unclear. This structuring might be limited as there is continuous gene flow between the two-chemotype populations, which may explain the chemotype shifts observed over the years in western Canada. The limited genetic structuring by trichothecene chemotype suggests populations that are not specific to either province or chemotypes might not be a very good indicators of population structure at the provincial level.

This study has provided current information on the population structure and chemotype diversity of *F. graminearum* isolates in wheat in three important wheat-producing provinces in Canada and on a state in the USA. The class B-Trichothecene profile of *F. graminearum* isolates world-wide recently reviewed by [52] is very variable in different regions. Management strategies should be geared towards chemotyping studies in local areas in order to correctly estimate and predicts risks in the face of climate changes and agronomic practices like crop rotation. Genetic analysis using VNTR data has revealed high levels of gene flow and genetic variation within the regions studied in the Provinces of Manitoba and Saskatchewan. The higher genetic diversity in Manitoba compared to Saskatchewan might result in more *F. graminearum* populations with better adaptation to current control strategies including resistant cultivars and fungicides. This is a result of constant sexual recombination between isolates that may suggest a drive towards more pathogenic *F. graminearum* isolates through mating. The *F. graminearum* isolates in this study were correlated with trichothecene chemotypes (3ADON and 15ADON) according to stringent genetic structure analyses. An understanding of the correlation of chemotype between or among populations and genetic structure would require more in-depth studies of thousands of isolates collected more recently from across Canada and the United States. In addition, this should contribute to the identification of any fitness attributes that might be associated with the frequency of chemotype shifts observed in this study. Finally, this information on genetic variation among subpopulations will contribute to genetic resistance breeding in wheat, as well as help in mycotoxin risk assessment and the monitoring the of FHB infected seed across Canada. 

## 4. Materials and Methods

### 4.1. Collection and Fungal Isolation

Wheat heads showing characteristic FHB symptoms were collected in 2018 and 2019 from the provinces of SK, MB and Ontario (ON), Canada and from the State of Pennsylvania (PA) in the northeastern USA. Twenty symptomatic wheat heads were collected from each location. Sampling was designed to cover as much of the geographic region as possible. Information on crop districts and locations sampled was recorded (Table S1). The symptomatic heads were collected randomly at each location and stored in paper envelopes. Infected kernels from the wheat heads were selected and surface sterilized in a 1% sodium hypochlorite solution for 1 min, rinsed in sterile distilled water and air-dried on sterile filter paper. Kernels were plated on potato dextrose agar (PDA; Difco Laboratories) and incubated at 25 °C for 7 days under fluorescent light. Pure cultures of *Fusarium* spp. were transferred onto Spezieller Nahrstoffarmer agar (SNA) media to allow sporodochia formation and further production of macroconidium cultures. These single spore isolates were stored in PDA media for further use. *Fusarium graminearum* isolates were identified based on morphological characteristics [53,54].

### 4.2. Genomic DNA Extraction and PCR Based Species Identification of F. graminearum

Genomic DNA was extracted according to Fernando et al. (2006). *Fusarium graminearum* isolates were confirmed using molecular markers (Fg16F and Fg16R) specific to *F. graminearum* [55], which produces an amplification of 450 bp. The PCR amplification reaction (25 µL) consisted of 20 ng template DNA, 2.5 µL 10× PCR buffer containing MgCl_2_ (FroggaBio, Concord, ON, Canada), 1 μL of dNTP (2.5 mM each), 0.25 μL of each primer (10 mM) and 0.5 U Taq DNA polymerase (FroggaBio, Concord, ON, Canada). Thermal cycling conditions consisted of an initial denaturation at 95 °C for 3 min; followed by 35 cycles of 30 s at 95 °C, 1 min at 56.7 °C, 1 min at 72 °C; and a final extension of 72 °C for 5 min. PCR products were run on a 1% agarose gel.

### 4.3. Trichothecene Genotype Identification

Multiplex PCR primers 3CON, 3NA, 3D3A and 3D15A [9] were employed to determine trichothecene chemotype of the *F. graminearum* isolates. This multiplex assay generates an 840 bp fragment for the NIV chemotype, 610 bp fragment for the 15ADON chemotype and 243 bp for the 3ADON chemotype. The PCR amplification reaction (25 µL) consisted of 20 ng template DNA, 2.5 µL 10× PCR buffer containing MgCl_2_ (FroggaBio, Concord, Canada), 1 μL of dNTP (2.5 mM each), 0.25 μL of each primer (10 mM) and 0.5 U Taq DNA polymerase (FroggaBio, Concord, Canada). The PCR thermal cycling conditions consisted of an initial denaturation at 94 °C for 4 min; followed by 35 cycles of 1 min at 94 °C, 40 s at 58 °C, 40 s at 72 °C; and a final extension of 72 °C for 6 min. PCR amplicons were separated on a 1.5 % agarose gel in 1× TAE buffer stained with RedSafe (FroggaBio, Concord, Canada) and sizes were estimated with a 100 bp DNA ladder.

### 4.4. Generation of VNTR Data

Isolate genotyping was performed with 10 VNTR markers (HK1043, HK913, HK913, HK957, HK965, HK967, HK1059, HK977, HK630, HK1073) developed by [30]. In total, 570 *F. graminearum* isolates were collected from the sampled wheat kernels in 2018 and 2019. All isolates were analysed by VNTR PCR. The PCR amplification reaction (25 µL) consisted of 20 ng template DNA, 2.5 µL 10× PCR buffer containing MgCl_2_ (FroggaBio, Concord, Canada), 2 μL of dNTP (2.5 mM each), 0.5 μL of each primer (10 mM) and 1.25 U Taq DNA polymerase (FroggaBio, Concord, Canada). PCR thermal cycling conditions consisted of an initial denaturation at 94°C for 10 min; followed by 35 cycles of 40 s at 94 °C, 40 s at 58 °C, 1 min at 72 °C; and a final extension of 72 °C for 10 min. The VNTR PCR products were visualised in high resolution 3% MetaPhor (Lonza, Basel, Switzerland) agarose gels. A 100 bp DNA ladder (New England Biolabs) was loaded in the gel and used to estimate band size. Isolates were clone-corrected in which isolates that have identical multilocus genotypes (identical alleles at all ten loci) were represented just once in each population. These resulted in 562 isolates used in the genetic structure analysis. *Fusarium graminearum* isolates with the same DNA band size were considered the same allele for each VNTR marker. The isolates were grouped into populations (provinces) and sub-populations (geographic regions within each province) based on geography (Figure 1; Table S1). Allelic sizes of all 10 markers estimated from bands observed on gels are listed in Table S1. Data of all 10 VNTR loci were combined to produce a multilocus genotype (G).

### 4.5. Population Genetic Analysis

Population genetic analysis of *F. graminearum* was performed to decipher population structure and determine the effects of geographic region and trichothecene genotypes. The provincial populations in Canada were divided into six geographic regions as sub-populations. We used GenAlEx v 6.5 [31,32] to estimate genetic diversity (*H*), Shannon’s information index (*I*), analysis of molecular variance (AMOVA), genetic distance based on ΦPT (a standardized equivalent of *F_ST_*), genetic identity and pairwise gene flow (*Nm*) for the geographic locations. The AMOVA determined the proportion of variation contributed by the geographic regions. The *Nm* was calculated based on ΦPT as *Nm* = 0.5[(1⁄ΦPT) − 1], where ΦPT was calculated as the proportion of the variance among populations relative to the total. We also determined the genetic variation between and within populations with AMOVA, by partitioning covariance components and their levels of significance (*p* < 0.05 or *p* < 0.01) using 1000 permutations. Genetic structure and admixture among populations were carried out with Bayesian clustering analyses of VNTR data using STRUCTURE v 2.3.4 [56]. We used the independent allele frequency and admixture (mixed ancestry) models in the analysis [56]. The number of simulated clusters (*K*) ranged from one to five after performing 100,000 Monte Carlo Markov Chain (MCMC) iterations following a 25,000-iteration burn-in for each run. Ten replicate runs were performed for each K value and STRUCTURE HARVESTER V. A.2 [57] was used to select the optimal model that maximized the rate of change in log-likelihood values (Δ*K*) based on the Evanno et al. (2005) method. The proportion of membership (*q*) in each of the K clusters was determined and isolates were assigned to the cluster that had the highest proportion of the membership.

## Figures and Tables

**Table 1:** Amplicon size range, number of alleles and allele frequencies of 10 VNTR markers of 570 *Fusarium graminearum* isolates analyzed in the study.

VNTR Locus ^a^	Chromosome	Amplicon Size Range	Total No. of Allelles	Allelic Frequency
HK1043	1	210–360	23	0.001–0.157
HK913	1	190–270	14	0.001–0.162
HK917	1	200–290	14	0.001–0.302
HK957	1	160–366	31	0.001–0.124
HK965	2	200–355	19	0.001–0.169
HK967	2	180–266	14	0.001–0.287
HK1059	3	190–290	15	0.001–0.169
HK977	3	180–300	17	0.002–0.266
HK630	6	190–300	18	0.001–0.220
HK1073	6	120–290	25	0.001–0.130

^a^ Locus name according to [30].

**Table 2:** Summary of genetic diversity statistics of *Fusarium graminearum* isolates from the Canada and northeastern USA.

	% of	N		Total							
Population	PL	2018	2019	Isolates	MLH		*N_a_*	*N_e_*	*I*	*H*	*uH*
SK1	100	58	0	58	58	Mean	9.000	5.925	1.834	0.789	0.803
						SE	1.116	0.841	0.140	0.037	0.038
SK2	100	68	38	106	103	Mean	13.800	7.677	2.193	0.847	0.856
						SE	1.356	1.203	0.122	0.017	0.017
MB1	100	62	109	171	168	Mean	17.200	9.176	2.387	0.879	0.885
						SE	1.569	1.075	0.101	0.011	0.011
MB2	100	58	30	88	88	Mean	15.400	9.679	2.390	0.883	0.893
						SE	1.681	1.280	0.114	0.012	0.012
MB3	100	26	0	26	26	Mean	8.800	6.234	1.921	0.819	0.852
						SE	0.772	0.669	0.109	0.022	0.023
ON	100	61	29	90	89	Mean	11.600	6.699	2.089	0.842	0.852
						SE	0.859	0.468	0.075	0.014	0.014
PA	100	0	31	31	30	Mean	8.600	5.692	1.841	0.800	0.827
						SE	0.718	0.640	0.116	0.025	0.026
Total		333	237	570	562	Mean	12.057	7.297	2.094	0.837	0.852
						SE	1.153	0.882	0.111	0.020	0.020

PL, Polymorphic loci N, No. of isolates within population, MLH, Number of multilocus haplotypes, *N_a_*, No. of observed alleles within the population, *N_e_*, No. of effective alleles, *I*, Shannon’s information index, *H*, Gene diversity, *uH*, Unbiased gene diversity; SE, Standard error of mean; SK1—Parkland region, Saskatchewan, SK2—southern Saskatchewan; MB1—southwest Manitoba, MB2—central Manitoba, MB3—Interlake region, Manitoba; ON—Ontario, PA—Pennsylvania, USA.

**Table 3:** Nei’s Genetic identity (above diagonal) and Genetic distance (below diagonal) of *Fusarium graminearum* isolates between the regions studied.

Population	SK1	SK2	MB1	MB2	MB3	ON	PA
SK1	****	0.817	0.772	0.766	0.670	0.547	0.430
SK2	0.202	****	0.889	0.859	0.752	0.680	0.534
MB1	0.259	0.118	****	0.877	0.752	0.718	0.609
MB2	0.267	0.152	0.131	****	0.774	0.716	0.581
MB3	0.401	0.286	0.285	0.256	****	0.470	0.416
ON	0.604	0.385	0.331	0.334	0.755	****	0.702
PA	0.844	0.627	0.496	0.542	0.877	0.354	****

SK1—Parkland region, Saskatchewan, SK2—southern Saskatchewan; MB1—southwest Manitoba, MB2—central Manitoba, MB3—Interlake region, Manitoba; ON–Ontario, PA—Pennsylvania, USA, ****—not applicable.

**Table 4:** Pairwise *F_ST_* values ^a^ (below diagonal) and pairwise gene flow ^b^ Nm (above diagonal) among Fusarium graminearum from the regions.

Population	SK1	SK2	MB1	MB2	MB3	ON	PA
SK1	****	10.279	9.027	8.583	4.971	4.699	3.038
SK2	0.024	****	21.686	16.852	7.214	7.889	4.227
MB1	0.027	0.011	****	21.750	7.590	9.756	5.232
MB2	0.028	0.015	0.011	****	7.927	9.464	4.828
MB3	0.048	0.033	0.032	0.031	****	3.829	2.803
ON	0.051	0.031	0.025	0.026	0.061	****	6.150
PA	0.076	0.056	0.046	0.049	0.082	0.039	****

^a^*F_ST_* value was calculated by 1000 randomizations by permutation of individuals among populations. ^b^ Gene flow (*Nm*) was calculated as *Nm* = 0.5[(1/ΦPT) − 1] with GenAlEx v. 6 [31,32], where ΦPT was calculated as the proportion of the variance among populations with respect to the total variance. SK1—Parkland region, Saskatchewan, SK2—southern Saskatchewan MB1—southwest Manitoba, MB2—central Manitoba, MB3—Interlake region, Manitoba; ON—Ontario, PA—Pennsylvania, USA, USA, ****—not applicable.

**Table 5:** Analysis of molecular variance of 562 clone corrected *Fusarium graminearum* isolates from five geographic regions in Saskatchewan and Manitoba, Canada.

Source of Variation	df	SS	MS	Est. Var.	%
Among populations	6	105.233	17.539	0.174	4%
Within populations	555	2394.785	4.315	4.315	96%
Total	561	2500.018		4.489	100%

**Table 6:** Assignment of 562 clone-corrected *Fusarium graminearum* isolates to the genetic clusters, CL1 and CL2 defined by STRUCTURE (*K* = 2) using 10 VNTR markers. The proportion of membership (*q*), of each cluster was used to assigned isolates in each geographic region to the clusters. Clusters with the most isolates are in bold.

Location	CL1 (Red)	CL2 (Green)	Total
Parkland region, Saskatchewan (SK1)	**52 (89.7%)**	6 (10.3%)	58
Southern Saskatchewan (SK2)	**73 (70.9%)**	30 (29.1%)	103
Southwest Manitoba (MB1)	**106 (63.1%)**	62 (36.9%)	168
Central Manitoba (MB2)	**57 (64.8%)**	31 (35.2%)	88
Interlake region, Manitoba (MB3)	**21 (80.8%)**	5 (19.2%)	26
Ontario (ON)	0 (0.0%)	**89 (100%)**	89
Pennsylvania, USA (PA)	0 (0.0%)	**30 (100%)**	30
Total no. of isolates	**309 (55%)**	**253 (45%)**	**562**

Clusters with the highest percentage in each region are indicated in bold.

**Table 7:** Trichothecene chemotype profile and genetic diversity parameters for the genetic clusters, CL1 and CL2 populations of 562 clone-corrected *Fusarium graminearum* analyzed by STRUCTURE (*K* = 2). Clusters with the highest number of isolates for each chemotype is in bold.

*K* ^a^	Isolates	*N_e_* ^b^	*I* ^c^	*H* ^d^	3ADON	15ADON
CL1	309	7.811	2.221	0.859	**239 (77.4%)**	70 (22.6%)
CL2	253	9.010	2.356	0.880	93 (36.8%)	**160 (63.2%)**

^a^*K*, population or cluster; ^b^*N_e_*, number of effective alleles; ^c^*I*, Shannon’s information index; ^d^*H*, unbiased gene diversity. Clusters with the highest percentage in each region are indicated in bold.

## Supplementary Materials

The following are available online at https://www.mdpi.com/2072-6651/13/3/180/s1, Table S1, Sample information for 570 *F. graminearum* isolates used in this study and the VNTR data obtained.
